# Clinical and Radiological Phenotypes and Endotypes

**DOI:** 10.1055/s-0041-1730894

**Published:** 2021-07-14

**Authors:** Ricardo J. José, Michael R. Loebinger

**Affiliations:** 1 Respiratory Medicine, Royal Brompton Hospital, London, United Kingdom; 2 Centre for Inflammation and Tissue Repair, University College London, London, United Kingdom; 3 National Heart and Lung Institute, Imperial College, London, United Kingdom

**Keywords:** bronchiectasis, phenotype, endotype, radiology, exacerbations

## Abstract

Bronchiectasis is a heterogenous disease with multiple etiologies and associated comorbidities. As bronchiectasis is a complex disease, it is unsound to think of it as a single disease particularly when the differing etiologies are likely to be driving bronchiectasis through initial divergent molecular pathways, known as endotypes, that phenotypically present as the same disease due to protracted airway inflammation, but revealing potential differing underlying mechanisms that may have disparity of drug responses. Improved understanding of the cellular immune, inflammatory, and microbiological milieu associated with clinical and radiological features of bronchiectasis has resulted in the recognition of important endotypes and phenotypes that will allow for personalized treatments to improve quality of life and outcomes of patients with bronchiectasis. Here we discuss clinical and radiological phenotypes, as well as emerging molecular endotypes that are possible treatable traits in bronchiectasis.

Bronchiectasis is defined radiologically as the abnormal widening of the bronchi and bronchioles within the lungs. This radiological feature is often associated with symptoms of chronic productive cough and recurrent respiratory infections leading to the clinical diagnosis of bronchiectasis. Although easily identified on computed tomography (CT) scans of the chest, clinically bronchiectasis is a complex disease with several known etiologies, associated comorbidities, and a heterogenous clinical course. Importantly, a proportion of patients continue to have bronchiectasis with an unidentified cause despite diagnostic efforts to establish an etiology. 1

In the last decade, there has been recognition by the medical and scientific community that bronchiectasis is a common respiratory condition with increasing prevalence and a lack of licensed therapeutics that have a significant impact on lung function decline, symptom burden, quality of life, and survival. The interest in bronchiectasis has led to efforts to better understand the pathogenesis of this disease, but it is a difficult task due to the etiological heterogeneity and it is not possible to identify these cases before bronchiectasis is established, except in the few cases where a genetic diagnosis is made at birth such as in cystic fibrosis. As bronchiectasis is a complex disease, it is therefore unsound to think of it as a single disease particularly when the differing etiologies are likely to be driving bronchiectasis through initial divergent molecular pathways, known as endotypes, that phenotypically present as the same disease due to protracted airway inflammation, but revealing potential differing underlying mechanisms that may have disparity of drug responses.

Research has therefore focused on understanding the cellular immune, inflammatory, and microbiological milieu associated with clinical and radiological phenotypes of bronchiectasis. The hope being that the identification of endotypes of bronchiectasis may translate into successful clinical trials of therapeutic interventions that to date have been hampered by the heterogeneity of bronchiectasis cases included in clinical trials, 2 and ultimately result in improved quality of life and outcomes for patients with bronchiectasis. Importantly, reductions in costs of genotyping are leading to greater use of this diagnostic test and identification of novel mutations that can be correlated with a clinical phenotype. This has been imperative in the identification of cystic fibrosis, primary ciliary dyskinesia, α1-antitrypsin deficiency, and immunodeficiencies. 3

Here, we discuss clinical and radiological phenotypes, as well as emerging molecular endotypes, that are possible treatable traits in bronchiectasis ( Fig. 1 ).

**Fig. 1:**
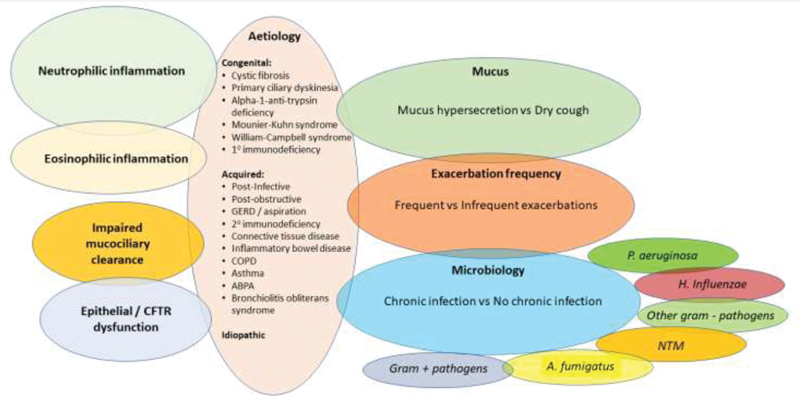
Bronchiectasis phenotypes/endotypes. CFTR, cystic fibrosis transmembrane conductance regulator; COPD, chronic obstructive pulmonary disease; NTM, nontuberculous mycobacteria.

## Clinical Phenotypes

Phenotype is the observable trait of an individual as a result of the individual's genetics, environment, or interaction of the two. In clinical practice, a phenotype is best described as the manifestations of a disease within an individual. This can be in relation to symptoms, associated comorbidities, frequency of exacerbations, disease severity, disease progression, and response to specific treatments. In simple terms, a specific genetic predisposition and environmental interaction would result in a precise phenotype, but in respiratory disease these interactions are complex and the resulting phenotype is not always reflective of the involvement of a specific molecular pathway, particularly when investigations of molecular pathways happen only after the disease is well established. Additionally, at this stage, molecular pathways may in turn be influenced by consequences of the disease such as following chronic infections and colonization with respiratory pathogens. Nevertheless, thorough assessment and investigations can assist in identifying molecular pathways that may predict disease progression and poor outcomes. Identifying phenotypes associated with disease progression and poor outcomes may allow for better characterization of individuals for treatment trials and these phenotypes may be amenable to interventions.

## Clinical Phenotype by Etiology

It is plausible that different causes of bronchiectasis may have a more notable molecular pathway involved in the disease process that could potentially be a unique target for treatments in these individuals. For example, in allergic bronchopulmonary aspergillosis (ABPA) with bronchiectasis, treatments target fungal sensitization with corticosteroids reducing IgE-mediated inflammation and antifungal agents decreasing airway fungal load and altering corticosteroid metabolism. In patients with antibody deficiency, immunoglobulin replacement therapy is given to correct the humoral defect and reduce sinopulmonary infections. The use of immunoglobulin replacement therapy seems to confer these patients a benefit; provided that patients with primary immunodeficiency receive immunoglobulin replacement, their disease course is similar to those with other causes of bronchiectasis. 4 5 However, it is important to note that in ABPA, the management of the fungal sensitization controls only one aspect of the disease and in primary immunodeficiency that despite receiving immunoglobulin replacement bronchiectasis may still develop and progress with lung function decline, 6 suggesting that correction of the defect related to the etiology of bronchiectasis is not always sufficient on its own to control the overall disease process. It is therefore important to recognize that within a phenotype there may be more than driver of exacerbations or disease progression. 7

A concern with using etiology as a suitable phenotype that will allow targeted intervention is that despite extensive assessment a cause for bronchiectasis is not always found and idiopathic bronchiectasis remains a common diagnosis. The proportion of patients without a known etiology depends on the cohort being investigated, and in specialist centers which perform systematic diagnostic evaluations, the incidence of idiopathic bronchiectasis is much lower. 8 Of the identified causes in specialist centers, postinfectious etiologies, chronic obstructive pulmonary disease (COPD), and causes of immune dysfunction are the most common. 8 9 Patients with postinfective bronchiectasis tend to be younger, while those with COPD tend to be older males with a smoking history. 10 Patients with idiopathic bronchiectasis are more likely to be female nonsmokers. 11

Studies investigating the impact of etiology on outcomes have included small number of participants and tend to group etiologies into postinfective bronchiectasis, non-postinfective bronchiectasis, and idiopathic bronchiectasis. 10 Such broad grouping of etiologies is not sufficient to identify unique phenotypes that may have specific associated endotypes or treatable traits. In larger studies where comparisons were made between more specific etiologies of bronchiectasis, there was not as strong association between cause of bronchiectasis and clinical outcomes, except in the two most prevalent groups in the study: COPD-associated bronchiectasis and idiopathic bronchiectasis. Participants with COPD-associated bronchiectasis were more likely to have severe disease and chronic infection with *Pseudomonas aeruginosa* and those with idiopathic bronchiectasis were more likely to have mild disease at the time of the study and not have chronic infection with *P. aeruginosa* . 10 12 In other studies, COPD patients with bronchiectasis have been shown to have increased disease severity, lung function decline, and hospitalizations but no difference in survival compared with COPD patients without bronchiectasis. 13 14 Despite the impact of COPD bronchiectasis on clinical outcomes, it is not clear if COPD is an actual cause of bronchiectasis or just an association. Furthermore, a study suggests that in COPD, the artery diameter is reduced and not that the bronchial diameter is larger, 15 suggesting possible pseudobronchiectasis.

Bronchiectasis has also been found in patients with asthma, particularly those with uncontrolled, severe, or steroid-dependent asthma. 16 However, the diagnosis of bronchiectasis in patients with asthma needs to be made with caution, because with good asthma control and reduction in airway inflammation these findings can be reversible. 17 Asthma-associated bronchiectasis is, however, an interesting phenotype because in most cases the asthma is driven by type 2, eosinophilic, and inflammation rather than the neutrophilic inflammation most commonly seen in other etiologies of bronchiectasis. This is therefore leading to new work looking at the role of eosinophils in bronchiectasis and attempts to target eosinophilic inflammation in these patients with agents other than corticosteroids. Although beneficial at reducing some types of airway inflammation, corticosteroid use in bronchiectasis may be associated with increased bacterial, mycobacterial, and fungal infections. A single-center study has shown that patients with a peripheral eosinophil count more than 300 cells/μL were more likely to have frequent exacerbations and worse quality of life. 18

Bronchiectasis has been shown to occur de novo in hematological malignancies and the mortality in this cohort is high. 5 19 Bronchiectasis in this group of patients is caused by acquired immunoglobulin deficiency, post–allograft stem cell transplantation, and in some cases rarer causes. Long-term observation and endotyping of the pulmonary inflammation and immunity in prospective studies of patients with hematological malignancy may identify important molecular pathways involved in the development of bronchiectasis.

Two important etiologies of bronchiectasis that are seen in clinical practice are connective tissue diseases and inflammatory bowel disease, particularly rheumatoid arthritis and ulcerative colitis, respectively. Patients with bronchiectasis and rheumatoid arthritis have been shown to have similar frequency of exacerbations and hospitalizations to patients with idiopathic bronchiectasis or other etiologies; however, they had higher mortality. 20 These are important etiology-associated phenotypes because their management will involve bronchiectasis treatment but also specific therapies to control the underlying inflammatory disease, usually with the use of immunosuppressant medication, or colectomy in the case of inflammatory bowel disease. Interestingly, cases have reported the development of bronchiectasis associated with inflammatory bowel disease after colectomy, which is usually curative of the inflammatory bowel in ulcerative colitis. 21 22

## Clinical Phenotype by Associated Microbiology

Chronic infection with *P. aeruginosa* has been shown to be an important driver of neutrophilic inflammation in bronchiectasis and has repeatedly been shown to be a phenotype associated with more severe disease, functional decline, frequent exacerbations and hospitalization, reduced quality of life, and decreased survival. 23 24 25 26 27 28

A meta-analysis comparing outcomes of patients colonized with *P. aeruginosa* to those without strongly demonstrated a sevenfold increased risk of hospital admissions and threefold increase in overall mortality, with 8% mortality at year 1 and 30 to 50% at 5 years. 24 Despite long-term awareness of the impact of *P. aeruginosa* infection on bronchiectasis, there is still no clear evidence on the management of first isolation of *P. aeruginosa* or the management of chronic *P. aeruginosa* infection with wide variation in practice among clinicians. 29 In cystic fibrosis, early eradication of *P. aeruginosa* is associated with improved outcomes such as reduced hospitalizations and lower rate of lung function decline 30 and failure of *P. aeruginosa* associated with increased frequency of exacerbations. 31 A small retrospective study showed 80% success of eradication of *P. aeruginosa* after first isolation, but this was short lived as approximately half of patients cultured *P. aeruginosa* again at 6 months. 32 Patients who had successful eradication had reduced frequency of exacerbations in the following year, but there was no difference in hospitalizations. 32 This is such an important area of bronchiectasis disease management, with significant impact on disease progression and quality of life, that research should be prioritized.

Other chronic infections are also associated with worse outcomes, with increased frequency of exacerbations, hospitalization, and mortality compared with those without chronic infection. 23 Colonization with *Haemophilus influenzae* has also been shown to be associated with increased frequency of exacerbations but without the need for hospitalization. 33

Nontuberculous mycobacteria (NTM) are isolated in patients with bronchiectasis with an estimated incidence of 2 to 14% 34 35 with *Mycobacterium avium* complex (MAC), including *M. avium* , *M. intracellulare* , and *M. chimaera* species, being the most common in bronchiectasis compared with other respiratory disease. The “Lady Windermere syndrome” is associated with NTM infections in elderly, underweight women with middle lobe bronchiectasis 36 37 ; however, in most patients with bronchiectasis, being underweight is an independent risk factor for NTM pulmonary disease. 38 In a study of NTM-pulmonary disease from South Korea with a high incidence of bronchiectasis, isolation of NTM did not result in changes to clinical indices of disease severity (forced expiratory volume in 1 second, body mass index, number of hospital admissions, and dyspnea score) but did lead to worsened radiological features. 35

## Clinical Phenotype by Symptoms

Symptoms are important because they are ultimately the manifestation of the disease during the individual's stable state and exacerbations. Furthermore, symptoms may be an expression of the etiology of bronchiectasis or complication. For example, those with asthma, COPD, or ABPA may have wheeze; those with aspiration may report symptoms of gastroesophageal reflux disease (GERD); and patients with mucus impaction may report chest pain. Although the main feature of bronchiectasis is a chronic productive “wet” cough, patients do not always produce large volumes of phlegm. Some may produce phlegm only during exacerbations. This latter group of bronchiectasis patients has lower levels of systemic inflammation and is less likely to have chronic infection. They also have lower severity and improved clinical outcomes. 23 In contrast, bronchiectasis patients who produce daily sputum are more likely to have levels of inflammation, disease severity, and clinical outcomes similar to those with chronic infections with pathogens other than *P. aeruginosa* . 23

## Clinical Phenotype by Exacerbation Frequency

Exacerbations are important events in the natural history of bronchiectasis. They are episodes of disease deterioration and important markers of disease activity, in those who have frequent exacerbations having more active diseases, poorer health status, worse quality of life, and increased mortality. 28 Furthermore, exacerbation frequency plays a particular key role on mortality. A large observational study has shown that although patients with chronic infection with *P. aeruginosa* have worse quality of life, mortality was increased only in the group that had at least two exacerbations per year. 39 As in COPD, prior exacerbations predict future exacerbations. 28 Reduction in exacerbation frequency is therefore an important goal of bronchiectasis management. Interventions that have been shown to reduce exacerbation frequency are macrolides, 40 inhaled antibiotics, 41 and pulmonary rehabilitation. 42

## Radiological Phenotypes

**Fig. 2:**
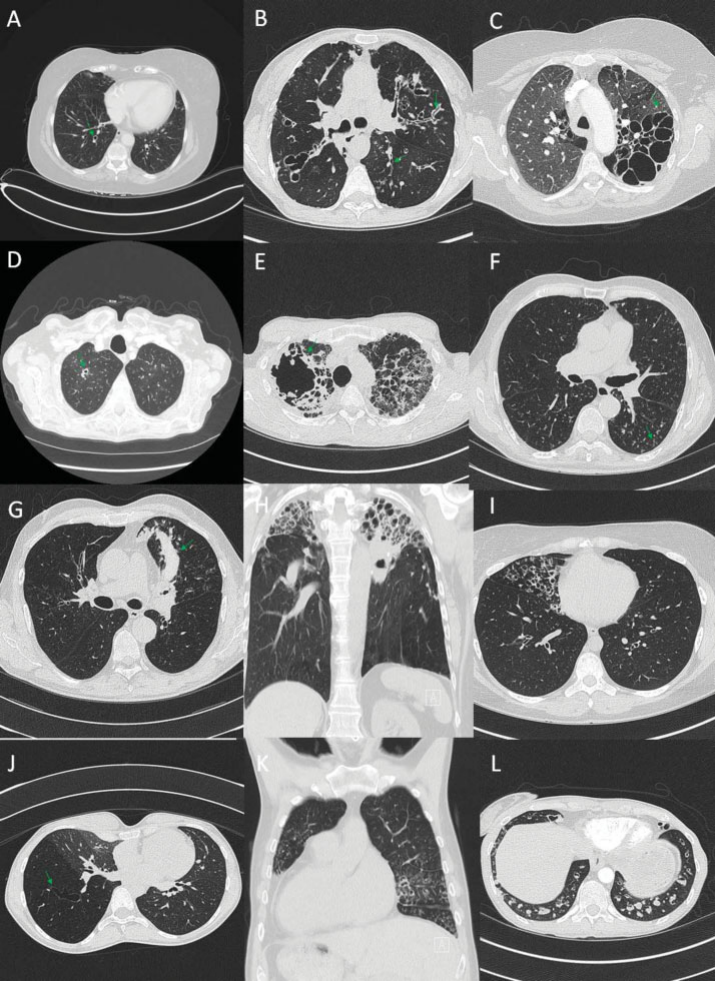
Radiological phenotypes. ( **A** ) Cylindrical bronchiectasis with signet ring sign. ( **B** ) Varicose bronchiectasis. ( **C** ) Cystic bronchiectasis. ( **D** ) Cavity in the right upper lobe. ( **E** ) Chronic pulmonary aspergillosis with right upper lobe cavitation with intracavitary material. ( **F** ) Tree-in-bud appearance. ( **G** ) Bronchocele in the left upper lobe in allergic bronchopulmonary aspergillosis. ( **H** ) Bilateral upper lobe bronchiectasis. ( **I** ) Isolated right middle lobe bronchiectasis. ( **J** ) Bronchiectasis in an area of hyperlucent lung with reduced vascularity (Swyer–James syndrome). ( **K** ) Situs inversus and bronchiectasis. ( **L** ) Mucus impaction of bronchiectatic airways.

Radiology imaging, especially CT scans of the chest, is paramount for the diagnosis of bronchiectasis. There are three predominant types of radiological patterns of bronchiectasis. In cylindrical bronchiectasis, the dilated bronchus has uniform caliber with lack of tapering of the distal airways ( Fig. 2A ). In varicose bronchiectasis, the dilated bronchus is not uniform and appears irregular and distorted ( Fig. 2B ). In cystic bronchiectasis, there is saccular dilatation of the bronchus with multiple saccular bronchi giving the appearance of cluster of cysts ( Fig. 2C ). Cavitation ( Fig. 2D ) can also be seen in bronchiectasis and may represent sequelae of previous pulmonary tuberculosis, NTM, pulmonary disease, or fungal infection (e.g., chronic pulmonary aspergillosis, Fig. 2E ). A very common finding is a tree-in-bud pattern due to mucus plugging of the distal small airways or infective bronchiolitis ( Fig. 2F ).

The distribution of bronchiectasis may also offer clues into the potential etiology. For example, central bronchiectasis is seen in ABPA ( Fig. 2G ), tracheobronchomegaly (Mounier–Kuhn syndrome); upper and middle lobe predominant bronchiectasis is seen in cystic fibrosis, previous TB, and ABPA ( Fig. 2H ); localized lobar bronchiectasis ( Fig. 2I ) may be due to bronchial obstruction (e.g., foreign body, endobronchial TB, or cancer), previous lobar pneumonia, aspiration, or Swyer–James syndrome (postinfectious bronchiolitis syndrome in childhood, Fig. 2J ); middle lobe bronchiectasis is often seen in Lady Windermere syndrome; and predominantly lower lobe bronchiectasis may be seen in primary ciliary dyskinesia, immunodeficiency, and α-1-antitrypsin deficiency. The finding of dextrocardia or situs inversus suggests PCD as a likely cause ( Fig. 2K ). It must, however, be noted that distribution of bronchiectasis is not pathognomonic for any specific cause. Furthermore, the finding of mucus impaction ( Fig. 2L ) is an indication for enhanced airway clearance and possibly the need for mucolytics.

## Endotypes

Endotypes are subtypes of a disease defined by distinct pathophysiological mechanisms and may allow treatment to be directed at specific pathways disordered within a given endotype. Due to the heterogeneity of bronchiectasis, the research community has started to look closer for molecular endotypes that may offer objective evidence of disease-specific biomarkers that may benefit a subpopulation of patients with bronchiectasis. It will also allow a better understanding. With improvements in techniques and reduction in costs, researchers can more readily analyze the individual's transcriptome, proteome, metabolome, and microbiome. The multiple, large collection of data can be integrated and analyzed with novel biological analysis approaches to determine relevant disease biomarkers and associations between entities—known as “multi-omics.”

Investigation of 83 individuals enrolled in the BAT (Bronchiectasis and Long-Term Azithromycin Treatment) study showed that the broad inflammatory profile was similar among individuals with bronchiectasis with asthma, COPD, or other and treated with or without azithromycin. 43 However, they did identify some differences among the co-diagnoses. For example, levels of IP-10/CXCL-10 distinguished between those with asthma and COPD/other. 43 Furthermore, in a stable state that sputum neutrophilic inflammation was lower in bronchiectasis patients infected with *P. aeruginosa* compared with those without *P. aeruginosa* infection. 43 Another group has shown that the combinations of microbiome and proteome sputum analysis can distinguish between individuals with COPD and bronchiectasis versus those individuals with COPD without bronchiectasis. 44 Those with COPD and bronchiectasis have a profile similar to that of bronchiectasis patients and is characterized predominantly by neutrophilic inflammation and proteobacteria dominant microbiome. From a separate report, it is demonstrated that in the majority (79%) of patients with bronchiectasis, the sputum proteome profile does not change between the stable and exacerbation state and is therefore linked to the baseline inflammatory state which in approximately 40% of individuals is neutrophilic. 45 An earlier study of 245 individuals, bronchiectasis had their sputum supernatant and serum analyzed for 21 potential biomarkers. 46 The researchers reported that principal component analysis identified three potential endotypes consisting of (1) eosinophilic and epithelial driven inflammation with elevated sputum levels of interleukin-5 (IL-5), IL-13, and Gro-α; (2) systemic inflammation with elevated serum levels of GMCF, IL-6, vascular endothelial growth factor, IL-10, and IL-1β; and (3) neutrophilic airway inflammation with elevated sputum levels of neutrophil extracellular traps (NETs), resistin, tumor necrosis factor, CXCL-8, IL-10, matrix metallopeptidase-9, and elastase. Individuals with predominantly neutrophilic inflammation had more severe disease and were more likely to be treated with inhaled antibiotics but overall, there were no significant difference in frequency of exacerbations among the three groups and no difference in outcomes in response to inhaled corticosteroid use or macrolide treatments. A recent study used liquid chromatography-tandem mass spectrometry to identify proteomic biomarkers associated with disease severity. 47 The majority of differentially expressed proteins identified were associated with neutrophilic inflammation, particularly components of NETs. NETs were associated with increased bronchiectasis severity and chronic bacterial infection. This study importantly showed that 14 days of antibiotic treatment had little effect on the NET-associated proteins in patients with chronic infection with *P. aeruginosa* , but that long-term macrolide treatment reduces NET and this immunomodulatory effect is important in those with *P. aeruginosa* infection, and is a favored agent to reduce exacerbation frequency and sputum volume.

## Future Directions

The last decade has seen an increase in interest and research activity in bronchiectasis. Prospective studies of large number of patients with bronchiectasis are needed to identify meaningful phenotypes and endotypes by using multi-omics techniques to combine data from several sources. These data should not just be the combination of datasets from different research groups worldwide or investigate specific cytokines/chemokines but instead employ novel investigation techniques using single-cell RNA sequencing, proteomics, metabolomics, and lipidomics combined with data regarding the microbiome, fungome, and virome, as well as clinical and physiological data in unbiased data analysis. Furthermore, although patients with bronchiectasis are usually diagnosed when they have symptoms and established disease, it will be important to look for patients with early disease and conduct long-term follow-up studies, and in some cases focus on at-risk groups to enable sampling prior to the development of bronchiectasis.
